# Anthocyanins accumulation analysis of correlated genes by metabolome and transcriptome in green and purple peppers (*Capsicum annuum*)

**DOI:** 10.1186/s12870-022-03746-y

**Published:** 2022-07-22

**Authors:** Yaning Meng, Hongxiao Zhang, Yanqin Fan, Libin Yan

**Affiliations:** Institute of Cash Crops, Hebei Academy of Agriculture and Forestry Sciences, Shijiazhuang, 050051 Hebei China

**Keywords:** Purple pepper, Anthocyanin, Enzyme, Transcription factor

## Abstract

**Background:**

In order to clarify the the molecular mechanism of anthocyanin accumulation in green and purple fruits of pepper using metabolomics and transcriptomics,to identify different anthocyanin metabolites*,*and to analyze the differentially expressed genes involved in anthocyanin biosynthesis..

**Results:**

We analyzed the anthocyanin metabolome and transcriptome data of the fruits of 2 purple pepper and 1 green pepper*.* A total of 5 anthocyanin metabolites and 2224 differentially expressed genes were identified between the green and purple fruits of pepper*.* Among the 5 anthocyanin metabolites,delphin chloride was unique to purple pepper fruits*,*which is the mainly responsible for the purple fruit color of pepper. A total of 59 unigenes encoding 7 enzymes were identified as candidate genes involved in anthocyanin biosynthesis in pepper fruit. The six enzymes (*PAL,C4H,CHI,DFR,ANS,UFGT*) had higher expression levels except the *F3H* gene in purple compared with green fruits. In addition,seven transcription factors were also found in this study. These transcription factors may contribute to anthocyanin metabolite biosynthesis in the fruits of pepper. One of differentially expressed gene novel.2098 was founded. It was not annotated in NCBI. Though blast analysis we preliminarily considered that this gene related to MYB transcription factor and was involved in anthocyanin biosynthesis in pepper fruit.

**Conclusions:**

Overall, the results of this study provide useful information for understanding anthocyanin accumulation and the molecular mechanism of anthocyanin biosynthesis in peppers.

**Supplementary Information:**

The online version contains supplementary material available at 10.1186/s12870-022-03746-y.

## Background

As living standards rise*,*more and more people begin to pay attention to their nutrition and healthcare*.* The development and utilization of anthocyanin compounds have become hot spots in the fields of phytochemistry*,* medicine*,*and healthcare*.* Anthocyanins*,*an important type of water-soluble pigment in plants*,*belong to the flavonoids and are widely distributed in plant organs,resulting in many colors including red*,*blue*,*and purple in plants [1, 2]. Anthocyanins have biological functions that improve health including antioxidation*,*anticancer*,*and antiaging functions*,*protecting eyesight*,*preventing cardiovascular diseases*,*and improving memory [3–5]. In addition*,* anthocyanins can reduce the photoinhibition of photosynthesis and photobleaching of chlorophyll under strong light*.* The accumulation of anthocyanins can increase the photostability of the photosynthetic system in seedlings*,*reduce plant tissue damage caused by high levels of Ultraviolet (UV) light,and improve stress resistance in plants [6].

Pepper (*Capsicum annuum* L.) is a plant that belongs to the genus Capsicum of the Solanaceae. There are many types of pepper germplasm resources*,*and purple pepper is one of the rarer types. Purple pepper is rich in anthocyanins and has good physiological tolerance to high temperature and drought stresses*.* Generally*,*plant fruit skin has the highest anthocyanin content*.* However*,*the fruit skin of most plants such as eggplant and purple sweet potato is inedible*,*whereas the edible part of purple pepper is the brightly colored pericarp*,*which is valuable for human health and directly determines its economic value and popularity in the market.

In recent years,progress has been made in the study of the regulation of anthocyanin biosynthesis and metabolism using genetics,genetic engineering,and molecular biology approaches. The biosynthesis of anthocyanins includes a series of metabolic reactions involving 20 different organic molecules and 12 different catalytic enzymes encoded by multiple homologous genes [7]. The biosynthesis of anthocyanins is an extension of the flavonoid pathway. Phenylalanine lyase (*PAL*),cinnamic acid 4-hydroxylase(*C4H*),and coumadin CoA ligase(*4CL*) are the starting points for the biosynthesis of flavonoids. The successive steps catalysed by the chalcone isomerase (*CHI*),flavonoid 3-hydroxylase(*F3H/FHT*),flavonoid 3′-hydroxylase(*F3’H*),dihydroflavonol-4-reductase (*DFR*),anthocyanidin synthase (*ANS*) and 3-glycosyltransferase(*3-GT*) enzymes lead to the production of the anthocyanin pigments. The late biosynthetic genes (*LBGs*) *F3’H*,flavonoid 3′5’-hyroxylase(*F3’5’H*),*DFR,ANS* and Anthocyanidin 3-O-glucosyl -transferase (*UFGT*) are essential for producing anthocyanin or some specific flavonoids [8–10].

In higher plants*,*the biosynthesis of most anthocyanins is often regulated in different spatial temporal patterns by combinations of multiple regulatory factors such as *R2R3-MYB,MADS-box,bHLH* and *WD40* [11, 12]. To date*,*several *R2R3-MYB* genes associated with either positive or negative regulation of the key genes (*DFR,ANS,*and *UFGT*) in anthocyanin biosynthesis have been identified*.* However*,*their contributions to the color of fruits and other organs vary [13–15]. *bHLH* transcription factor is also a key regulator of anthocyanin biosynthesis*,*usually independently regulating *CHS,DFR,*and *UFGT* [16]. *WD40* proteins do not bind to the promoters of the anthocyanin biosynthetic genes. Instead*,*they interact with *bHLH* and *MYB* to regulate anthocyanin biosynthesis [17]. The *R2R3MYB,bHLH,*and *WD40* proteins usually form a transcriptional activation complex (*MYBbHLH-WD40,MBW*) to regulate the transcription of late anthocyanin biosynthetic genes in most plants [18–20].

In recent years, Metabolomics and transcriptomics have been widely used to study the biosynthesis of metabolites and their molecular mechanisms [21, 22]. The research on the regulation mechanism of anthocyanin synthesis has been reported in *capsicum annuum* [23–26], but different materials of purple pepper contained different anthocyanin metabolites, so the molecular mechanism of anthocyanin biosynthesis was slightly different. Therefore, In this study,2 purple and 1 green pepper (check, CK) were analyzed by means of targeted metabolome and transcriptome technologies to discover the differential genes of enzyme and transcription factors associated with anthocyanin synthesis in peppers, To explore the molecular mechanism of purple pepper fruit formation, provide theoretical and technical support for expanding purple pepper gene resources and promoting purple pepper molecular breeding.

## Results

### Analysis of pepper anthocyanin metabolome

**Fig. 1:**
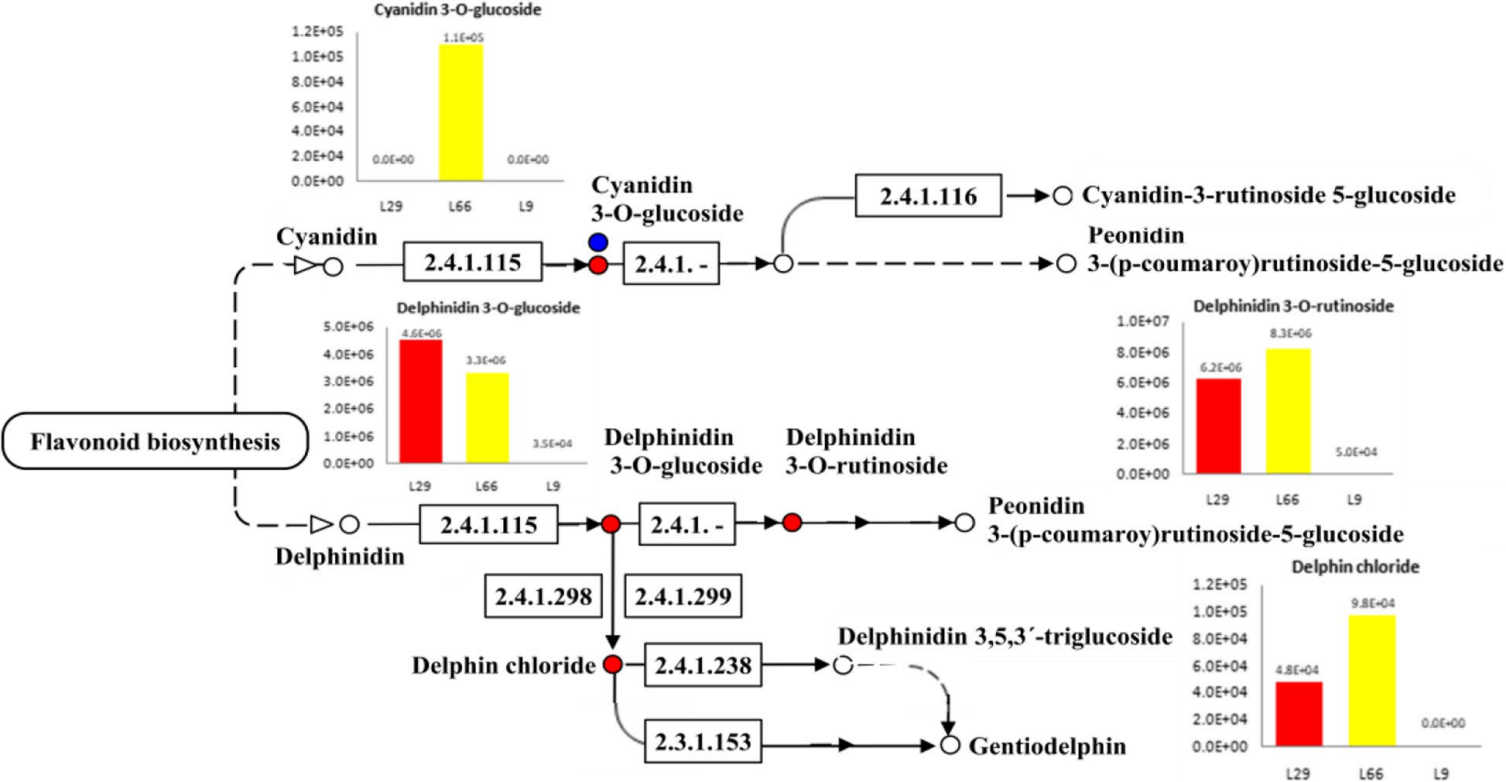
Schematic diagram of pepper anthocyanin differential metabolite relative content and pathway. Note: The red circle indicates that the metabolites are significantly upregulated; The blue circle indicates that the metabolites are not changed. The histogram shows the relative contents of different metabolites of anthocyanins

Anthocyanidins are the most important flavonoid pigments in plants. In order to compare the composition of anthocyanin metabolites between three pepper, L66,L29,and L9(CK),the anthocyanin-like substances in pepper fruits were examined. Principal component analysis (PCA) showed that the samples between the groups scattered and the samples within the groups clustered,indicating that the anthocyanin metabolome data was reliable. On the principal component PC1 and PC2,L9(CK) was clearly separated from L29 and L66 and the difference was significant (Fig. S1). A total of 5 anthocyanin like compounds were detected in the fruits of three pepper. The contents of delphinidin 3-O-glucoside,delphinidin 3-O-rutinoside,and delphin chloride in L66 and L29 were significantly higher than L9(CK). Delphin chloride was found in the fruits of purple pepper L66 and L29,but not in L9(CK). This suggesting that it is a unique metabolite in purple pepper fruits. Delphin chloride could be mainly responsible for the purple fruit color of pepper. The cyanidin 3-O-galactoside content in the fruits of the green pepper line was higher than that in the purple pepper L29 fruits,indicating that the green pepper fruit also accumulated anthocyanins to a certain extent (Fig. 1). The KEGG database was used to annotate the differential metabolites. No annotation result was obtained for cyanidin 3-O-galactoside. The rest of the detected metabolites were found to share the metabolic pathway ko00942.It can be seen from the KEGG pathway map that three of the 4 annotated metabolites were significantly upregulated in purple pepper(L29,L66) and they were delphinidin-derived products.

### Statistics of purple and green pepper transcriptome sequencing data

We further investigated the differences in gene expression among the three samples*.* With three biological replicates*,*the transcriptome sequencing of the 9 samples yielded a total of 63.26Gb clean data with 94.14% of bases scoring Q30 (Table S1)*.* The transcriptome sequencing reads were aligned with the reference genome with efficiencies ranged from 86.14 to 95.04% (Table S2), which showed a normal rate of data utilization,suggesting that the selected reference genome was suitable for subsequent analysis.

### DEGs between purple and green peppers and KEGG enrichment analysis

**Fig. 2:**
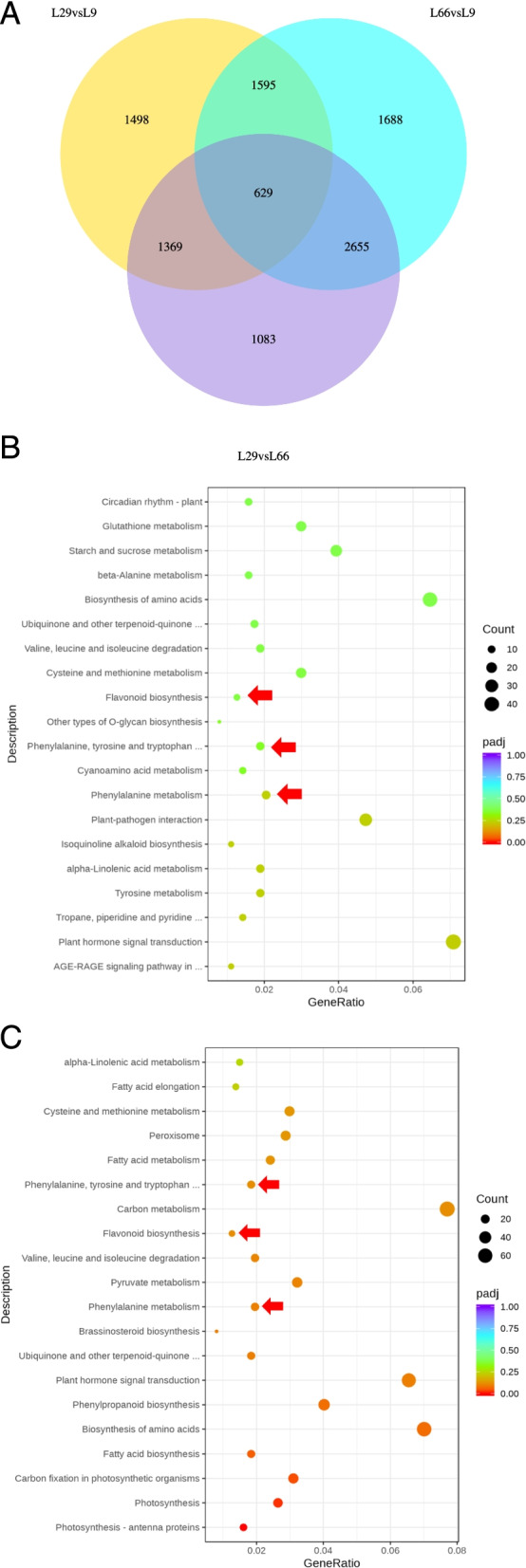
Venn diagram of differentially expressed genes (DEGs) and scatter plots of KEGG enrichment in 3 pepper. **A** Venn diagram of DEGs in 3 pepper. **B** KEGG enrichment scatter plot of L66 vs. L9. **C** KEGG enrichment scatter plot of L29 vs. L9*.*Red arrows indicate: the three KEGG pathways related to anthocyanin. The red arrow from top to bottom in Fig. **B**:Phenylalanine, tyrosine and tryptophan, Flavonoid biosynthesis, Phenylalanine metabolism; The red arrow from top to bottom in Fig. **C**: Flavonoid biosynthesis, Phenylalanine, tyrosine and tryptophan, Phenylalanine metabolism

In order to clarify DEGs and their biological pathways between purple and green peppers*,*we analyzed the Tr*ANS*criptome sequencing between the fruits of 2 purple pepper (L66 and L29) and 1 green pepper (L9). The number of DEGs were 6567 (L66 vs. L9) and 5091 (L29 vs. L9)*.* A total of 2224 DEGs were common in both of the purple pepper (Table S3). The 2224 DEGs were annotated and 111 KEGG pathways were found. Eight of the top 20 KEGG pathways were shared by the two purple pepper,of which 3 pathways (ko00360,ko00400,and ko00941) were anthocyanin-related (Fig. 2).

### Regulation of anthocyanin biosynthetic pathway genes in purple and green pepper

**Fig. 3:**
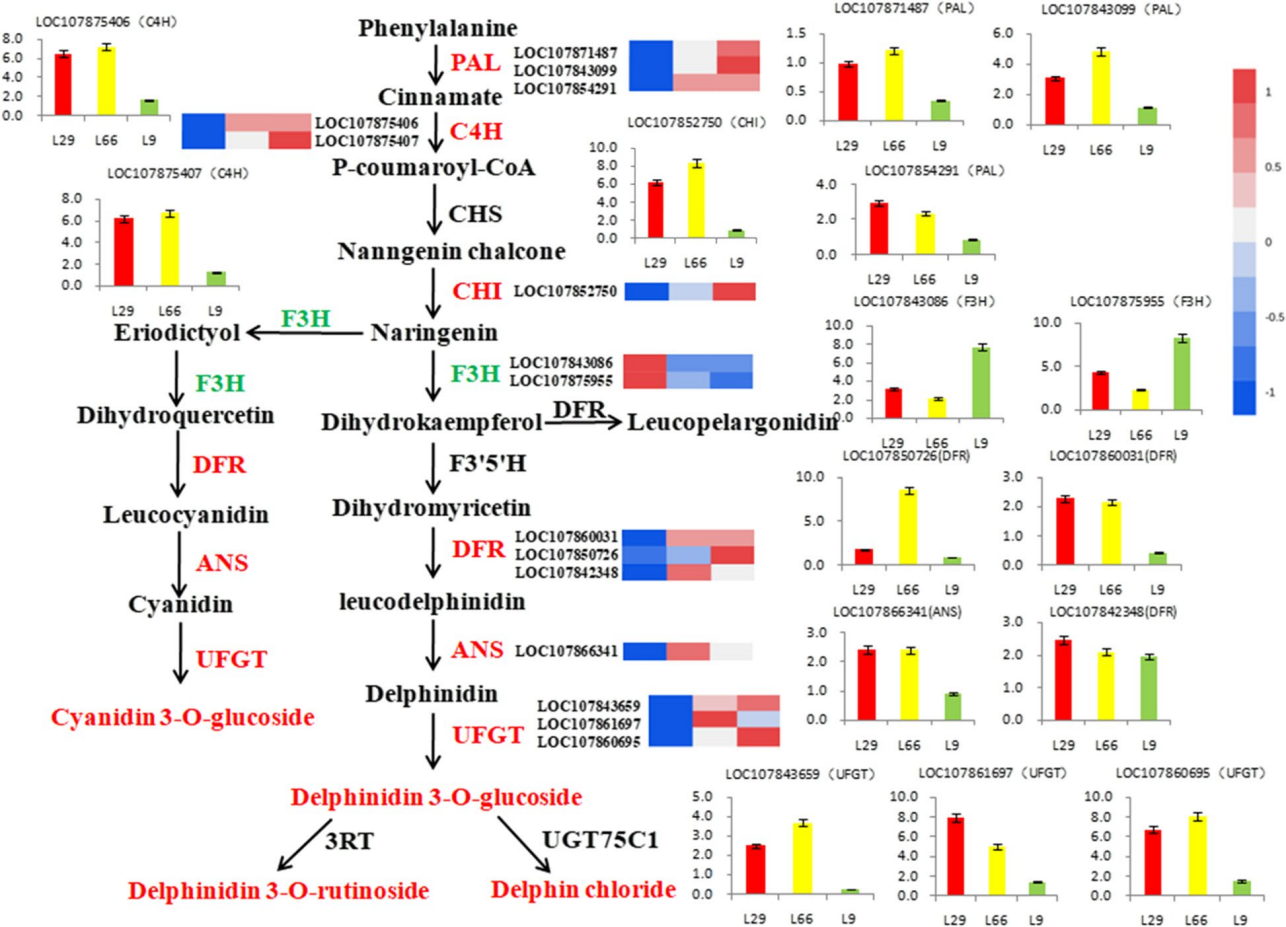
Heat map of anthocyanin biosynthesis pathway and differential expression of enzyme gene in Pepper*.* Red color denotes a significant up-regulation*.* Green color denotes a significant down-regulation

Anthocyanins are one of the natural products synthesized through the metabolic pathways of phenylpropanes and flavonoids*.* We analyzed the anthocyanin biosynthesis pathway in order to performed the key genes in the metabolism of purple and green fruits pepper. In total*,*59 unigenes that encoded 9 enzymes in the anthocyanidin biosynthesis pathways were studied. Fifteen unigenes of the seven enzymes had different expression levels,including 13 upregulated and 2 downregulated unigenes (Fig. 3). The six enzymes (*PAL*、*C4H*、*CHI*、*DFR*、*ANS*、*UFGT*) had higher expression levels except the *F3H* gene in purple compared with green fruits*.* The 13 unigenes were upregulated by 1.04 to 13.13 fold (Log 2 FC) and 2 unigenes were downregulated by − 1.5 to − 3.5 fold (Table S4).

### qRT-PCR validation

We examined differential expression of unigenes between purple and green peppers to identify which unigenes had expression correlating with anthocyanin. The expression levels of anthocyanin biosynthetic genes *PAL* (LOC107871487*,*LOC107843099*,*LOC107854291)*,C4H*(LOC107875407,LOC107875406),*CHI* (LOC107852750), *F3H*(LOC107843086,LOC107875955),*DFR* (LOC107850726,LOC107860031,LOC107842348),*ANS* (LOC107866341),and *UFGT* (LOC107843659*,*LOC107861697*,*LOC107860695) were determined by RT-qPCR using leaves of both purple pepper(L66,L29) and green pepper(L9)*.* The results showed that the expression levels of the 6 genes were significantly higher in fruits of purple pepper than that in fruits of the green pepper line*,*but The expression of two genes in *F3H* was higher in green pepper*.* The qRT-PCR results of the expression of 15 genes were highly consistent with the results obtained based on transcriptome sequencing (Fig. 3), indicating that DEG analysis was highly reliable.

### Transcription factors regulating anthocyanin biosynthesis in pepper fruits

**Fig. 4:**
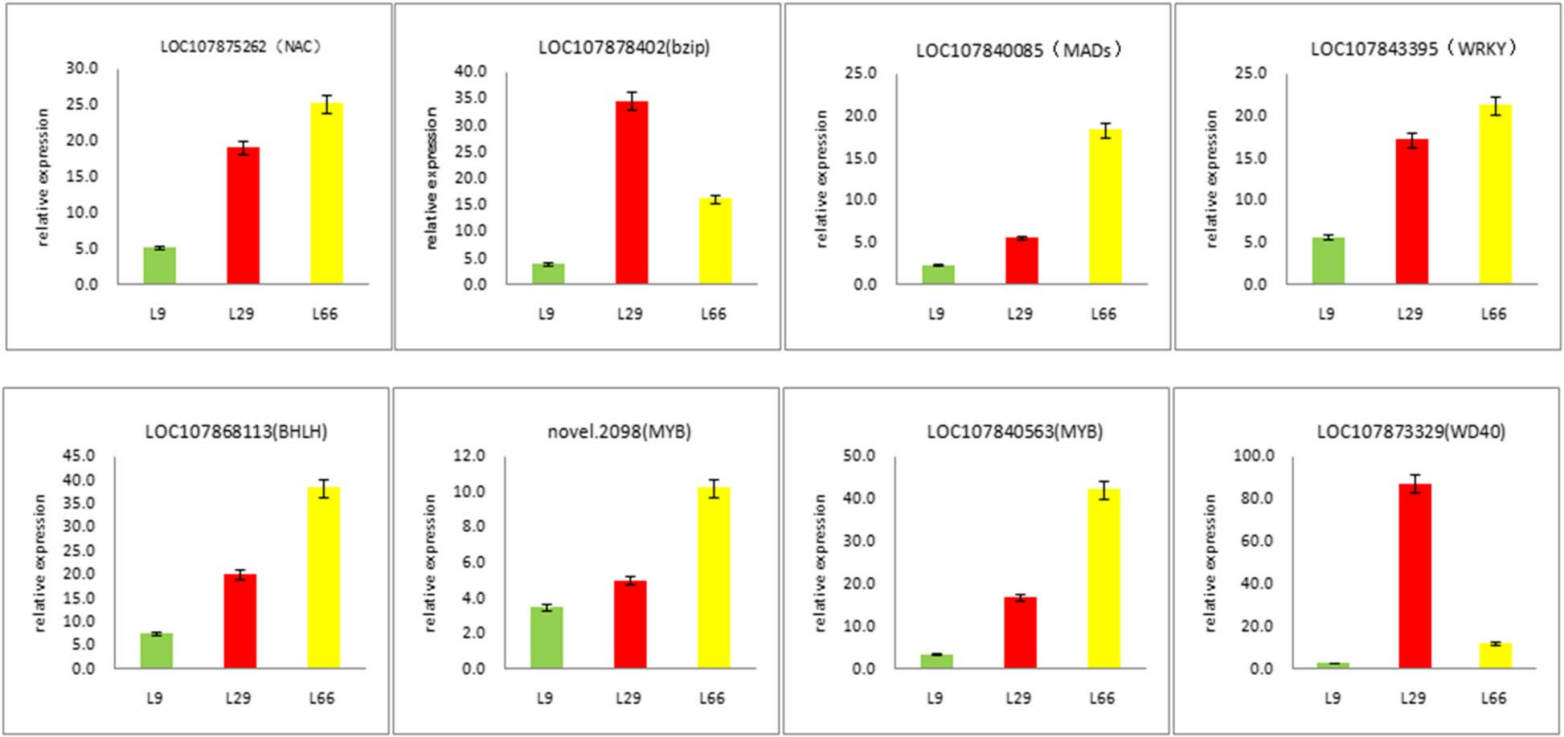
Expression analysis of 8 transcription factor genes during anthocyanin biosynthesis in purple pepper and green pepper fruits

Transcription factors (TF) are special genes that modulate the expression levels of other genes and represent the main players for the determination of spatiotemporal transcriptional activity [27]. Among the annotated unigenes*,*transcription factors related to anthocyanin synthesis were MYB,*bHLH,WD40,Bzip,WRKY,MA-DS, Zinc-finger* and *NAC.* In the same transcription factor*,* DEGs in the two kinds of purple pepper are not identical. All DEGs encoding eight TFs were retrieved and the results revealed that *bHLH*,*MYB* and *NAC* were the more DEGs which may differentially regulate structural genes participating in the Anthocyanin formation of pepper (Table S5). Most of these TFs were found up-regulated in purpel peppers(L66 and L29) when compared to the green pepper(L9)*,*suggesting that a high transcriptional activity is required to form the anthocyanin (Table 1)*.* Eight genes were randomly selected from each transcription factor for QRT-PCR verification*,*and their expression trends were highly consistent with RNA-SEQ results*,*indicating that DEG analysis was highly reliable (Fig. 4, Table S6).

**Table 1:** Transcription factors of anthocyanin biosynthesis in fruit of *Capsicum annuum*

Gene	Enzyme	L29 vs L9			L66 vs L9		
		No*.*All DEGs	No*.*Up DEGs	No.Down DEGs	No*.*All DEGs	No*.*Up DEGs	No.Down DEGs
*MYB*	MYB TF	47	26	21	63	45	18
*bHLH*	Basic helix-loop-helix protein	33	23	10	38	30	8
*WD40*	WD40 repeat protein	12	8	4	19	13	6
*NAC*	NAC domain containing protein	42	38	4	32	29	3
*WRKY*	WRKY DNA-binding protein	21	11	10	28	17	11
*MADs*	MADS-box TF	24	16	8	25	9	16
*Zinc-finger*	Zinc finger protein	1	0	1	3	3	0
*Bzip*	Basic leucine zipper	4	2	2	5	3	2

No.All DEGs, the total number of DEGs

No.Up DEGs, the number of upregulated DEGs

No.Down DEGs, the number of downregulated DEGs

### The gene involved in anthocyanin biosynthesis in fruits of *Capsicum annuum*

**Fig. 5:**
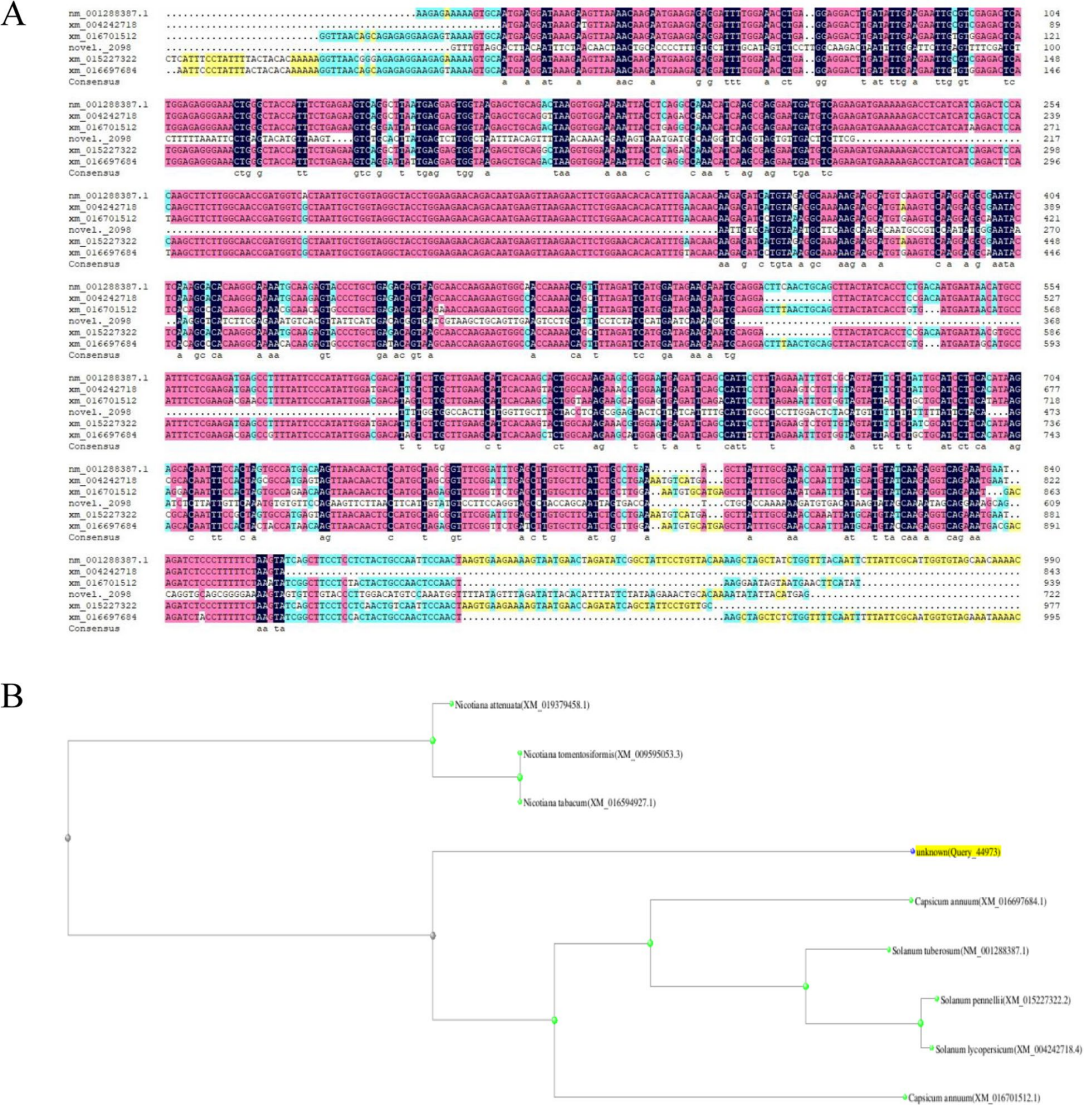
The homology alignment analysis of novel.2098 in different species. **A** Blastn analysis; **B** Phylogenetic tree of novel.2098 in different species. Unknown: represents annotated with novel.2098

During the biosynthesis of anthocyanin in *capsicum annuum*,we found a differentially expressed gene novel*.*2098 that was not annotated in NCBI*.* Blast analysis indicated that the novel.2098 gene exhibited 86.48,84.75 and 84.24% amino acid identity to NM_001288387.1(*Solanum tuberosum*),XM_016701512.1 (*Capsicum annuum*) and XM_015227322.2 (*Solanum pennellii*). Phylogenetic analysis revealed that the novel.2098 gene had a close relationship with the *MYB* protein of *Solanum tuberosum*. Though blast analysis we preliminarily considered that this gene related to *MYB* transcription factor and was involved in anthocyanin biosynthesis in pepper fruit (Fig. 5).

## Discussion

### Anthocyanin identification in the fruits of *Capsicum annuum*

Purple pepper had been gaining more popularity because of their antioxidant function,antiaging effects and attractive colors. Anthocyanin as one of the important pigment play a crucial role in the formation of pepper fruit color [28]. The anthocyanin accumulation causes pepper fruit to appear purple and black [29]. According to the studies reported previously,delphinidin is the only anthocyanidin found in peppers [30]. In our study, The accumulation levels of these anthocyanins in particular,delphinidin 3-O-glucoside,delphinidin 3-O-rutinoside and delphin chloride positively correlated with the purple flesh coloration,implying that these compounds partly contribute to the purple color. Among the above three delphinidins,delphin chloride was unique in purple pepper. This result that delphin chloride could be mainly responsible for the purple fruit color of pepper.

### Structure genes associated with anthocyanin biosynthesis in fruits of *Capsicum annuum*

Anthocyanidin biosynthesis belongs to a branch pathway of flavonoids that starts from phenylalanine. After carboxylation,glycosylation,methylation,and acylation,the anthocyanins are transported to and accumulated in vacuoles. A variety of enzymes participate in the biosynthesis of anthocyanins in higher plants [31, 32]. It has been reported that there was no significant difference in the expression levels of *PAL*,*C4H*,CHS and *4CL* between purple and green peppers,while the expression levels of *F3H*, *F3’5’H*,*DFR*,*ANS*,and *UFGT* were all significantly higher in purple pepper than in green peppers [33–35].However,it was also suggested that the high expression of *PAL*, *C4H* and *4CL* in purple pepper might be one of the reasons for the high anthocyanin content [29]. In our study,we found that the expressions of CHS and *4CL* did not change significantly in purple pepper fruits, while the expressions of *DFR*,*ANS*,*CHI*,*PAL*,*C4H* and *UFGT* were significantly increased during anthocyanin biosynthesis and the expression level of *F3H* gene was low in purple pepper fruit, which was slightly different with the results of previous studies. Therefore, we think preliminarily *DFR*,*ANS*,*CHI*,*PAL*,*C4H* and *UFGT* play a positive regulatory role in anthocyanin biosynthesis,and their high expression is conducive to the production of anthocyanin derivatives. However,*F3H* gene was low expressed in purple pepper,and its decrease did not affect the increase of delphinidin products,and played a role in reverse regulation of anthocyanin biosynthesis. The structural genes of anthocyanin biosynthesis have been studied a lot,but the results are slightly different,which may be caused by different experimental materials and environmental conditions.

### Transcription factor associated with anthocyanin biosynthesis in fruits of *Capsicum annuum*

In addition to structural genes*,*transcription factors are also the key factors in the synthesis of anthocyanins in plants*.* To date*,*transcription factors of *MYB,bHLH,WD40,NAC,zinc finger, MADs* and *WRKY* proteins have been found to regulate anthocyanin biosynthesis [29, 36–39]. In peppers*,*through qRT-PCR and RNA-Seq analysis*,* Tang et al. [40] found that two *MYBs* (*CaANT1* and *CaANT2*)*,*one *bHLH* (*CaAN1*)*,*and one *WD40*(CaTTG1) transcription factor participated in the accumulation of anthocyanins in pepper flowers*,*and might activate anthocyanin accumulation by forming a new *MBW* complex*.* In recent studies,it reported that the WRKY transcription factors interacts with the *MBW* complex regulate the anthocyanin biosynthetic pathway [34] and some *MADS-box* proteins have involved in the synthesis of flavonoids [41]. It was speculated that *NAC* and *H2C2* transcription factors may regulate the expression levels of these *MYB/HD-Zip* genes in the ripening periods [42]. In this study,we found that transcription factors related to anthocyanin synthesis of pepper were *MYB,bHLH,WD40,Bzip,WRKY,MADS,Zinc-finger* and *NAC*. Among them*,*transcription factors *bHLH NAC* and *MYB* were the more DEGs which may differentially regulate structural genes participating in the anthocyanin formation of pepper. At present*,*many transcription factors have been found in the process of anthocyanin biosynthesis in pepper*,*but the relationship between transcription factors is not clear*,*which needs further research and verification.

Furthermore,In this study*,*we detected one differentially expressed *MYB* genes (novel.2098) that were upregulated in green vs purple pepper*.* Blast analysis indicated that the novel.2098 gene is a new MYB gene in *Capsicum annuum*. According to the results of the forefathers [43, 44], we speculated that the high expression of the *MYB* gene stimulated the upregulation of structural gene expression, which led to the accumulation of anthocyanin derivatives. Therefore,the high expression level of the *MYB* gene may have stimulated the formation of anthocyanin, leading to the formation of purple fuirts.

By analyzing the transcriptome and metabolome data of purple and green pepper*,*we found that the biological metabolism of anthocyanin is complex and affected by multiple transcription factors and enzyme genes*.* Different gene expression levels lead to changes in the accumulation of anthocyanin metabolites,resulting in different degrees of purple in pepper. This experiment studied the metabolic pathways and key genes for the color of purple pepper fruits,which provided new views into the synthesis and accumulation of anthocyanins in pepper fruits and laid a foundation for more effective cultivation of purple pepper. In addition,the biosynthesis of pepper anthocyanins is not only affected by the key enzyme genes and transcription factors,but also affected by various biochemical reactions and environmental conditions. More in-depth research is needed in the future.

## Conclusions

Metabolome and transcriptome data were used to reveal the anthocyanin biosynthesis metabolic pathway in pepper fruit. Changes in anthocyanin metabolites,delphinidin 3-O-glucoside and delphinidin 3-O-rutinoside contribute to the purple color. Delphin chloride could be mainly responsible for the purple fruit color of pepper. In an analysis of the anthocyanin biosynthesis pathway,we identified 59 unigenes encoding 7 enzymes that are involved in anthocyanin biosynthesis in fruits of pepper. In addition,seven transcription factors were also found that could contribute to anthocyanin metabolite biosynthesis in the fruits of pepper. Our results provide valuable information on the anthocyanin metabolites and the candidate genes involved in the anthocyanin biosynthesis pathways in pepper.

## Methods

### Plant materials

**Fig. 6:**
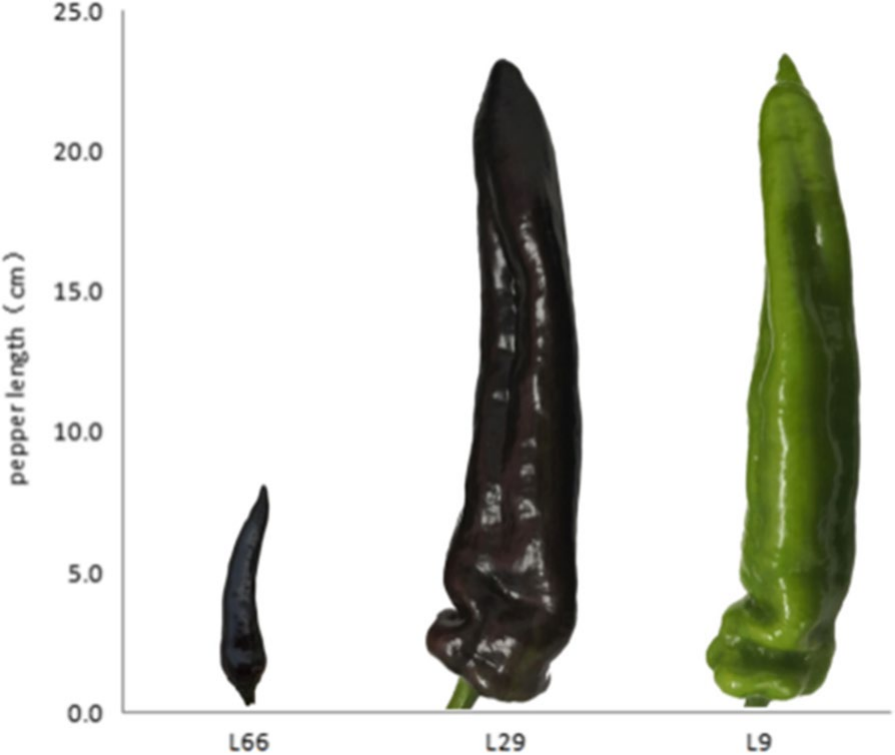
The phenotype of purple and green pepper fruits

Two purple pepper L66 and L29 and a green pepper line L9 were provided by the Economic Crop Research Institute of Hebei Academy of Agricultural and Forestry Sciences*.* Purple pepper line L66 fruits have deeper purple color than L29 fruits (Fig. 6)*.* The pepper were sown in seedling trays in early January and transplanted. Pericarp tissues of the matured fruits collected from different plants were sampled (each line had three repeats)*,*frozen in liquid nitrogen*,*and then stored at − 80 °C for subsequent metabolite extraction*,*transcriptome sequencing (RNA-seq)*,*and real-time quantitative PCR (qRT-PCR) analysis.

### Metabolite extraction and high-performance liquid chromatography (HPLC) conditions

Multiple reaction monitoring (MRM) was performed by Metware Biotechnology (Wuhan*,C*hina)*.*Freeze-dried leaves were crushed using a mixer mill (MM 400*,*Retsch) with a zirconia bead for 1.5 min at 30 Hz*.*Then*,*100 mg of the powder was extracted overnight at 4 °C with 1.0 mL 70% aqueous methanol*.* Following centrifugation at 10,000 g for 10 min*,*the extracts were absorbed and filtrated (SCAA-104,0.22 μm). The sample extracts were analyzed using an LC-ESI-MS/MS system (HPLC*,*Shim-pack UFLC Shimadzu CBM30A systemMS*,*Applied Biosystems 4500QTRAP)*.* The analytical conditions were as follows:high-performance liquid chromatography (HPLC): column*,* waters ACQUITY UPLC HSS T3 C18 (1.8 μm,2.1 mm*100 mm);solvent system*,*water (0.04% acetic acid): acetonitrile(0.04% acetic acid);gradient program*,*100:0 V/V at 0 min*,*5:95 V/V at 11.0 min*,*5:95 V/V at 12.0 min*,*95:5 V/V at 12.1 min*,*and 95:5 V/V at 15.0 min;flow rate*,*0.40 mL/min; temperature*,*40 °C;injection volume:5 μL*.* The effluent was alternatively connected to an ESI-triple quadrupole-linear ion trap (Q TRAP)-MS [45, 46].

### ESI-q trap-MS/MS

Linear ion trap (LIT) and triple quadrupole (QQQ) sc*ANS* were acquired on a triple quadrupole-linear ion trap mass spectrometer (Q TRAP), API 6500 Q TRAP LC/MS/MS System*,*equipped with an ESI Turbo Ion-Spray interface*,*operating in a positive ion mode and controlled by Analyst 1.6 software (AB Sciex)*.* The ESI source operation parameters were as follows:ion source*,*turbo spray*,*with source temperature at 500 °C;ion spray voltage (IS),5500 V; ion source gas I (GSI)*,*gas II (GSII)*,*and curtain gas (CUR) at 55*,*60*,*and 25.0 psi,respectively; and the collision gas (CAD) was 12 psi*.*Instrument tuning and mass calibration were performed with 10 and 100 μmol/L polypropylene glycol solutions in QQQ and LIT modes*,*respectively*.* QQQ scans were acquired as MRM experiments with the collision gas (nitrogen) set to 5 psi*.* Declustering potential (DP) and collision energy (CE) for individual MRM transitions were done with further DP and CE optimization*.* A specific set of MRM transitions were monitored for each period according to the metabolites eluted within that period.

### RNA extraction and Illumina sequencing

Total RNAs for constructing nine cDNA libraries of the three pepper*,*each with three replicates*,*were isolated from frozen pericarp tissues using the modified CTAB method [47]. mRNAs were used as templates for reverse transcription into cDNAs that were then used to construct cDNA libraries after purification*.* Agilent 2100 bioanalyzer was used to detect the insert size to control the quality of the libraries*.* The qualified libraries were sequenced on the Illumina HiSeq 4000 platform*.* The reads of adapters*,* N-containing sequences (N indicates ambiguous bases)*,*and low-quality sequences (reads with the bases that have Qphred 20 or less accounting for over 50% of the entire read length) were removed*.* HISAT2 software was used to quickly and accurately align the clean reads with the reference genome (https://www.ncbi.nlm.nih.gov/genome/term=Capsicum%20annuum) to locate the reads on it*.* FPKM (Fragments Per Kilobase of transcript per Million fragments mapped) was used to correct the sequencing depth and gene length [48]. DESeq software [49] was employed to analyze and screen the genes differentially expressed between sample groups*,*adopting |log_2_(Fold Change)| > 1 and padj< 0.05 as the screening standard*.* The differentially expressed genes (DEGs) were then analyzed through GO enrichment and KEGG [50] functional annotation of metabolic pathways.

### Verification of DEGs by qRT-PCR

Real-time fluorescence qRT-PCR was used to verify the results obtained based on transcriptome sequencing*.* Primer Premier 5.0 software (Primer*,*Canada) was used to design specific primers for DEGs*.* The PCR program was as follows: 95 °C for 5 min followed by 40 cycles of 95 °C for 15 s and 60 °C for 30 s*.* The PCR was repeated 3 times for each sample*.* Actin (serial number: GQ339766) was used as the internal reference gene to determine the relative expression level of DEGs.

## Supplementary Information

**Additional file 1: Fig S1.** Principal component analysis (PCA) of two purple (L66,L29) and one green (L9) pepper fruits.

**Additional file 2: Table s1.** Summary of sample sequencing data quality.

**Additional file 3: Table s2.** Sample and reference genome comparison statistics.

**Additional file 4: Table S3.** Results of gene expression in purple vs green peppers.

**Additional file 5: Table S4.** Differentially expressed 15 structural genes of anthocyanin biosynthesis in purple vs green peppers.

**Additional file 6: Table S5.** Differentially expressed 8 transcription factors of anthocyanin biosynthesis in purple vs green peppers.

**Additional file 7: Table S6.** Differentially expressed genes of transcription factors in purple vs green peppers.

## Data Availability

All data generated or analysed during this study are included in this published article and its Supplementary information files. The raw RNA-seq data are freely available at: http://www.ncbi.nlm.nih.gov/bioproject/ PRJNA827972.

## Abbreviations

Dp: Delphinidin
DFR: Dihydroflavonol 4-reductase
ANS: Anthocyanidin synthase
UFGT: Anthocyanidin 3-O-glucosyltransferase
CHS: Chalcone synthase
CHI: Chalcone isomerase
F3H: Flavonone 3-hydroxylase
F3’H: Flavonoid 3′-monooxygenase
F3’5’H: Flavonoid 3′,5′-hydroxylase
UGT75: Anthocyanidin 3-O-glucoside 5-O-glucosyltransferase
BHLH: Basic helix-loop-helix
DEGs: Differentially expressed genes
UPLC: Ultra- performance liquid chromatography
LIT: Linearion hydrazine-flight time
QQQ: Triple quadrupole
GSI: Gas I
GSII: Gas II
CUR: Curtain gas
CAD: Collision gas
MRM: Multiple reaction monitoring
DP: Declustering potential
CE: Collision energy
PCA: Principal component analysis
KEGG: Kyoto Encyclopedia of Gene and Genome
NCBI: National Center for Biotechnology Informatio
GO: Gene Ontology
qRT-PCR: Quantitative real-time polymerase chain reaction
UV: Unit variance scaling

## References

[1] Liu Y, Tikunov Y, Schouten R (2018). Anthocyanin biosynthesis and degradation mechanisms in solanaceous vegetables: a review. Front Chem.

[2] Naing A, Kim C (2018). Roles of *R2R3-MYB* transcription factors in transcriptional regulation of anthocyanin biosynthesis in horticultural plants. Plant Mol Biol.

[3] Marszalek K, Wozniak L, Kruszewski B, Skapaka S (2017). The effect of high pressure techniques on the stability of anthocyanins in fruit and vegetables. Int J Mol Sci.

[4] Yousuf B, Gul K, Wani A, Singh P (2016). Health benefits of anthocyanins and their encapsulation for potential use in food systems. Crit Rev Food Sci Nutr.

[5] Wong MK, Takei Y (2013). Lack of plasma kallikrein-kinin system cascade in teleosts. PLoS One.

[6] Nakabayashi R, Yonekura Sakakibara K, Urano K, Suzuki M, Yamada Y, Nishizawa T, Matsuda F, Kojima M, Sakakibara H, Shinozaki K, Michael A, Tohge T, Yamazaki M, Saito K (2014). Enhancement of oxidative and drought tolerance in Arabidopsis by overaccumulation of antioxidant flavonoids. Plant J.

[7] Nakatsuka T, Nishihara M, Mishiba K (2005). Two different mutations are involved in the formation of white flowered gentian plants. Plant Sci.

[8] Aza-Gonzalez C, Herrera-Isidron L, Núñez-Palenius HG, Martínez De La Vega O, Ochoa-Alejo N (2013). Anthocyanin accumulation and expression analysis of biosynthesis-related genes during chili pepper fruit development. Biol Plant.

[9] Zhang Z, Lid W, Jin H, Yin YX, Zhang HX, Chai WG, Gong ZH (2015). VIGS approach reveals the modulation of anthocyanin biosynthetic genes by *CaMYB* in chili pepper leaves. Front Plant Sci.

[10] Passeri V, Koes R, Quattrocchilio FM (2016). New challenges for the Design of High Value Plant Products: stabilization of Anthocyanins in plant vacuoles. Front Plant Sci.

[11] Hichri I, Barrieu F, Bogs J, Kappel C, Delrot S, Lauvergeat V (2011). Recent advances in the transcriptional regulationof the flavonoid biosynthetic pathway. J Exp Bot.

[12] Brounp (2005). Transcriptional control of flavonoid biosynthesis: a complex network of conserved regulators involved in multiple aspects of differentiation in Arabidopsis. Curr Opin Plant Biol.

[13] Kobayashi S, Yamamoto NG, Hirochika H (2016). Association of VvmybA1 gene expression with anthocyanin production in grape (Vitis vinifera) skincolor mutants. Hortic Sci.

[14] Strygina KV, Kochetov AV, Khlestkina EK (2019). Genetic control of anthocyanin pigmentation of potato tissues. BMC Genet.

[15] Niu SS, Xu CJ, Zhang WS, Bo Z, Xian L, Linwang K, Chen KS (2010). Coordinated regulation of anthocyanin biosynthesis in chinese bayberry (Myricarubra) fruit by a R2R3-MYB transcription factor. Planta..

[16] Stommel JR, Lightbourn GJ, Winkel BS, Griesbach RJ (2009). Transcription factor families regulate the anthocyanin biosynthetic pathway in Capsicum annuum. J Hortic Sci.

[17] Dumm JM, Stommel JR (2015). Coordinated regulation of biosynthetic and regulatory genes coincides with AnthocyaninAccumulation in Developing Eggplant Fruit. Hortic Sci.

[18] Zhao L, Gao L, Wang H, Chen X, Wang Y, Yang H, Xia T (2013). The *R2R3-MYB,bHLH,WD40,*and related tr*ans*cription factors in flavonoid biosynthesis. Funct Integr Genomics.

[19] Docimo T, Francese G, Ruggiero A, Batelli G, Depalma M, Bassolino L, Toppino L, Rotino GL, Mennella G, Tucci M (2016). Phenylpropanoids accumulation in eggplant fruit:characterization of biosynthetic genes and regulation by a *MYB* transcription factor. Front.Plant..

[20] Montefiori M, Brendolise C, Dare AP, Lin-Wang K, Davies KM, Hellens RP, Allan AC (2015). In the Solanaceae,a hierarchy of bHLHs confer distinct target specificity to the anthocyanin regulatorycomplex. J Exp Bot.

[21] Jiang T, Zhang M, Wen C, Xie X, Tian W, Wen S, Lu R, Liu L (2020). Integrated metabolomic and transcriptomic analysis of the anthocyanin regulatory networks in *Salvia miltiorrhiza* Bge*.*flowers. BMC Plant Biol.

[22] Cai X, Lin L, Wang X, Xu C, Wang Q (2018). Higher anthocyanin accumulation associated with higher transcription levels of anthocyanin biosynthesis genes in spinach. Genome..

[23] Jung S, Venkatesh J, Kang MY, Kwon JK, Kang BC (2019). A non-LTR retrotransposon activates anthocyanin biosynthesis by regulating a MYB transcription factor in Capsicum annuum. Plant Sci.

[24] Liu J, Ai X, Wang Y, Lu Q, Li T, Wu L, Sun L, Shen H (2020). Fine mapping of the Ca3GT gene controlling anthocyanin biosynthesis in mature unripe fruit of Capsicum annuum L. Theor Appl Genet.

[25] Guoab Y, Baiab J, Duanab X, Wangab J (2021). Accumulation characteristics of carotenoids and adaptive fruit color variation in ornamental pepper. Sci Hortic.

[26] Villa-Rivera MG, Ochoa-Alejo N (2021). Transcriptional Regulation of Ripening in Chili Pepper Fruits ( Capsicum spp.). Int J Mol Sci.

[27] Banerjee N, Zhang MQ (2003). Identifying cooperativity among transcription factors controlling the cell cycle in yeast. Nucleic Acids Res.

[28] Lightbourn GJ, Griesbach RJ, Novotny JA, Clevidence BA, Rao DD, Stommel JR (2008). Effects of anthocyanin and carotenoid combinations on foliage and immature fruit color of Capsicum annuum L. J Hered.

[29] Liu Y, Lv J, Liu Z, Wang J, Yang B, Chen W, Lijun O, Dai X, Zhang Z, Zou X (2020). Integrative analysis of metabolome and transcriptome reveals the mechanism of color formation in pepper fruit (*Capsicum annuum* L.). Food Chem.

[30] Aza-Gonzalez C, Nuez-Palenius HG, Ochoa-Alejo N (2012). Molecular biology of chili pepper anthocyanin biosynthesis. Chem Sci.

[31] Zhang Q, Su LJ, Chen JW (2012). The antioxidative role of anthocyanins in Arabidopsis under high irradiance. Biol Plant.

[32] Tingrui MA, Wen J, Guang ZH (2012). Anthocyanin synthesis and gene regulation. Agricult Sci Technol.

[33] Zhang H, Zhao X, Zhang J, Yang B, Yu Y, Liu T, Nie B, Song B (2020). Functional analysis of an anthocyanin synthase gene StANS in potato. Sci Hortic.

[34] Borovsky M, Oren-Shamir R, Ovadia WD, Jong I (2004). The a locus thatcontrols anthocyanin accumulation in pepper encodes a *MYB* transcription factorhomologous to Anthocyanin2 of Petunia. Theor Appl Genet.

[35] Jung S, Venkatesh J, Kang M-Y, Kwon J-K, Kang B-C (2019). A non-LTR retrotransposon activates anthocyanin biosynthesis by regulatinga MYB transcription factor in Capsicum annuum. Plant Sci.

[36] Gonzalez CA, Isidron LH, Palenius HG (2012). Anthocyanin accumulation and expression analysis of biosynthesis related genes during chill pepper fruit development. Biol Plant.

[37] Schaart JG, Dubos C, Romero I (2013). Bovy*.* Identification and characterization of *MYB-bHLH-WD40* regulatory complexes controlling proanthocyanidin biosynthesis in strawberry (Fragaria 9 ananassa) fruits. New Phytol.

[38] Lloyd A, Brockman A, Aguirre L, Campbell A, Bean A, Cantero A, Gonzalez A (2017). Advances in the *MYB-bHLH-WD* repeat (*MBW*) pigment regulatory model: addition of a *WRKY* factor and cooption of an anthocyanin MYB for Betalain regulation. Plant Cell Physiol.

[39] Xin N, Yang Z, Li Z, Yi T, Wang J, Zhiping Z (2015). Mechanisms of *MYB-bHLH-WD40* complex in the Regu-lation of anthocyanin Biosynthes. Agricult Biotechnol.

[40] Tang B, Li L, Hu Z, Chen Y, Tan T, Jia Y, Xie Q, Chen G (2020). Anthocyanin accumulation and transcriptional regulation of anthocyanin biosynthesis in purple pepper. J Agric Food Chem.

[41] Nesi N, Debeaujon I, Jond C, Stewart AJ, Jenkins GI, Caboche M, Lepiniec L (2002). The transparent testa16 locus encodes the arabidopsis bsister *MADS* domain protein and is required for proper development and pigmentation of the seed coat. Plant Cell.

[42] Zhang Q, Wang L, Liu Z, Zhao Z, Zhao J, Wang Z, Zhou G, Liu P, Liu M (2020). Transcriptome and metabolome profiling unveil the mechanisms of *Ziziphus jujuba* Mill*.*peel coloration. Food Chem.

[43] Gomez-Maldonado J, Avila C (2004). Functional interactions between a glutamine synthetase promoter and *MYB* proteins. Plant J.

[44] Duthie G, Crozier A (2000). Plant-derived phenolic antioxidants. Curr Opin Lipidol.

[45] Zhang S, Ying H, Pingcuo G, Wang S, Zhao F, Cui Y, Zeng X. Identification of potential metabolites mediating bird’s selective feeding on Prunus mira flowers. Biomed Res Int. 2019;(2019):1–8. 10.1155/2019/1395480 PMC6612375 31341887

[46] Zhang X, Jiang X, He Y, Li L, Xu P, Sun Z, Li J, Xu J, Xia T, Hong G (2019). AtHB2 a class II HD-ZIP protein, negatively regulates the expression of Csans which encodes a key enzyme in *Camellia sinensis* catechin biosynthesis. Physiol Plant..

[47] Chai GH, Qi G, Cao YP, Wang ZG, Li Y, Tang XF, Yu YC, Wang D, Kong YZ, Zhou GK (2014). Poplar PdC3H17 and PdC3H18 are direct targets of PdMYB3 and PdMYB21 and positively regulate secondary wall formation in Arabidopsis and poplar. New Phytol.

[48] Bray N, Pimentel H, Melsted P (2015). Near-optimal RNA-Seq quantification. Xiv preprint.

[49] Anders S, Huber W (2010). Differential expression analysis for sequence count data. Genome Biol.

[50] Kanehisa M, Furumichi M, Sato Y, Ishiguro-Watanabe M, Tanabe M (2021). KEGG: integrating viruses and cellular organisms. Nucleic Acids Res.

