# Supplementary figures and images for: Anthocyanins accumulation analysis of correlated genes by metabolome and transcriptome in green and purple peppers (Capsicum annuum)
Source: BMC Plant Biol. 2022 Jul 22;22:358. doi: 10.1186/s12870-022-03746-y (PMC9308287; doi:10.1186/s12870-022-03746-y)

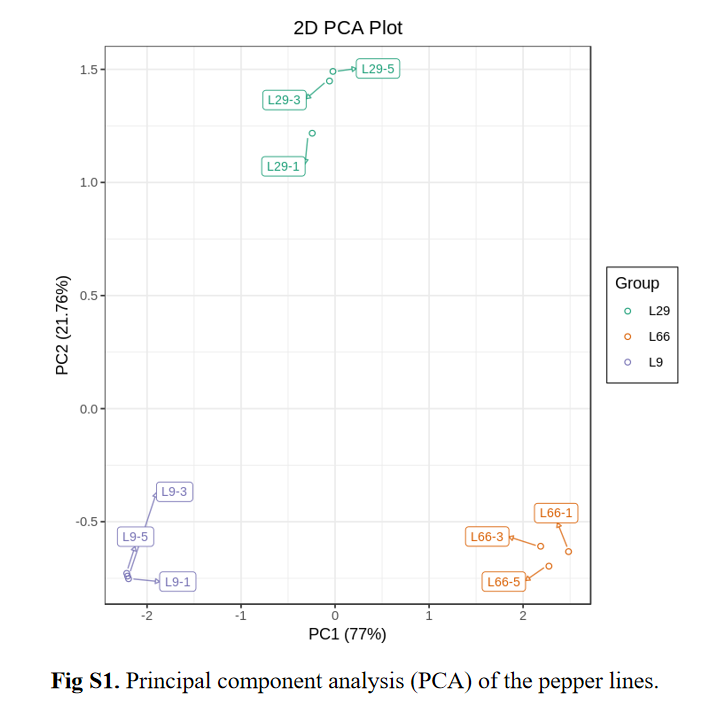

Supplement: Supplementary file 1 — Additional file 1: Fig S1. Principal component analysis (PCA) of two purple (L66,L29) and one green (L9) pepper fruits. [file 12870_2022_3746_MOESM1_ESM.png]
